# A Clinical Evaluation of the Safety, Efficacy, and Tolerability of the Soulflower Rosemary Redensyl Hair Growth Serum, Tetragain™, in Healthy Female Subjects for the Treatment of Alopecia: Promoting Hair Growth and Reducing Gray Hair

**DOI:** 10.7759/cureus.77066

**Published:** 2025-01-07

**Authors:** Maheshvari Patel, Natasha Tuli, Nayan Patel, Apeksha Merja

**Affiliations:** 1 Clinical Research, NovoBliss Research Private LImited, Ahmedabad, IND; 2 Pharmacology, Swaminarayan University, Kalol, IND; 3 Research and Development, PT Invent India Private Limited, Mumbai, IND

**Keywords:** alopecia, gray hairs, hair density, hair fall, hair growth

## Abstract

Introduction

Alopecia and graying hair, common conditions influenced by aging, genetics, and environmental factors, often see limited success with traditional treatments. Rosemary extract, valued for its antioxidant and anti-inflammatory benefits, has gained attention for promoting hair growth and reducing graying. Redensyl™, a plant-based ingredient, supports hair follicle regeneration and enhances hair density. This study examines the effectiveness of Soulflower Rosemary Redensyl Hair Growth Serum (Tetragain™), which is formulated using Redensyl, *Oryza sativa* (rice) water extract , *Salvia hispanica* (chia) seed extract, rosemary oil, MelanoGray™, and AnaGain™, in promoting hair growth, enhancing follicular activity, and reducing graying.

Methods

This open-label, single-arm, prospective interventional study evaluated the safety, efficacy, and tolerability of a hair growth and anti-gray hair serum. Ethical approval was obtained, and participants provided informed consent. The study measured changes in hair growth rate, length, density, thickness, anagen:telogen (A:T) ratio, hair fall, graying severity, and scalp appearance using a phototrichogram (CASLite Nova, Catseye Systems & Solutions Pvt Ltd, Navi Mumbai, Maharashtra, India), the 60-second hair comb test, and dermatological evaluations. Consumer perception of the test treatment was evaluated using a questionnaire. Statistical analysis was conducted using IBM SPSS Statistics for Windows, Version 29.0.1.0 (Released 2023; IBM Corp., Armonk, New York, United States) and Microsoft Excel 2019 (Microsoft Corporation, Redmond, Washington, United States), with results reported at a 5% significance level.

Results

The study found that applying a test treatment led to a significant reduction in gray hairs and an improvement in hair growth over the study duration of 120 days after the use of the test treatment. Hair growth rate was enhanced by 46.71% on Day 90, hair length improved by 35.40% on Day 87, A:T Ratio improved by 48.26% on Day 90, hair density improved by 37.92%, hair thickness improved by 80.85% on Day 120, reduction in hair fall observed by 64.89% on Day 120, improvement in Graying Severity Score improved by 64.89% after the use of the test treatment, which was statistically significant (p-value of <0.001). The experience of the test was reported by all the subjects as 'effective' in improving hair growth and gray hairs and they were satisfied after use. No adverse effects were observed during the study.

Conclusion

The test treatment, Soulflower Rosemary Redensyl Hair Growth Serum (Tetragain), exhibited a favorable safety profile, with no reported adverse reactions, making it suitable for regular use in daily hair care routines. Its formulation effectively enhances hair density, reduces hair fall, and addresses early signs of graying, providing a clinically supported option for individuals seeking to improve hair health and maintain natural color. This formulation can normalize the hair growth cycle, thereby fostering an optimal environment for sustained hair growth.

## Introduction

Alopecia refers to hair loss that can affect the scalp or other areas of the body, with common types including androgenetic alopecia, alopecia areata, and telogen effluvium. It is characterized by hair thinning, patchy bald spots, or complete hair loss. Symptoms can include increased shedding, receding hairlines, or smooth, hairless patches. The hair growth cycle is disrupted, causing shorter, finer strands or a delay in regrowth. Depending on the type and cause of alopecia, treatments may vary, ranging from topical medications like minoxidil to systemic therapies aimed at restoring the natural hair growth cycle and preventing further loss [1].

Androgenetic alopecia (AGA) comprises male pattern hair loss (MPHS) and female pattern hair loss (FPHL). FPHL is the most prevalent cause of hair thinning in women, characterized by a progressive reduction in hair density, particularly over the central scalp and crown. Unlike MPHL, FPHL typically spares the frontal hairline and leads to diffuse thinning rather than complete baldness [2]. 

Gray hair is one of the signs of aging, resulting from a decline in melanin production. Melanin is the pigment that gives hair its colorand is synthesized by melanocytes, specialized cells located within the hair follicles. As people age, these melanocytes progressively decrease in activity or eventually die off, leading to diminished melanin levels. Consequently, hair loses its original color, transitioning to shades of gray, silver, or white [3]. 

Hair follicles are influenced by hormonal changes, genetics, age, and environmental stressors. Androgenetic alopecia, autoimmune diseases, and nutritional deficiencies can disrupt hair growth cycles [4]. Pathogenesis of hair loss involves dysregulation of the hair cycle, inflammation, and follicular miniaturization. Genetic predisposition, hormonal imbalances, oxidative stress, and immune system dysfunction are key factors in hair growth disruption [5,6]. Premature graying, a natural age-related process, is characterized by the gradual loss of pigmentation in hair shafts [7,8]. Premature graying may indicate underlying medical conditions like thyroid disorders, vitamin B12 deficiency, or autoimmune diseases. Stress, smoking, and oxidative damage also contribute to the graying process. Understanding the molecular mechanisms underlying hair growth offers new opportunities for targeted interventions [9].

Research on treatments for gray hair focuses on restoring or enhancing melanin production through antioxidant and anti-aging therapies. Factors like melanocyte stem cell depletion, oxidative damage, and reduced melanin synthesis contribute to graying. Understanding these molecular mechanisms offers potential avenues for innovative treatments [10]. Common treatments for promoting hair growth and addressing gray hair typically include natural ingredients like vitamin B3, saw palmetto, coconut oil, and rosemary oil [11]. However, there are several other ingredients that have been shown to provide benefits.

Redensyl™ (Givaudan S.A., Vernier, Switzerland), a patented ingredient, supports hair follicle regeneration and reduces hair loss [12]. It is a hair growth ingredient that targets hair follicle stem cells and human fibroblasts to promote hair regeneration and reduce hair loss. Its blend of patented molecules, including dihydroquercetin glucoside (DHQG) and glycine, enhances hair follicle stem cell activity, increasing hair density and reducing shedding. Clinical studies show it stimulates new hair growth, improves hair strength, and may prevent premature graying [13].

*Oryza sativa* (rice) water extract [14] and *Salvia hispanica* (chia) seed extract [15] are beneficial for nourishment and hydration, while *Rosmarinus officinalis* (rosemary) oil supports scalp health and stimulates hair follicles, while caffeine enhances hair shaft elongation [16]. Keratin and biotin strengthen hair structure, while *Melaleuca alternifolia* (tea tree) oil reduces dandruff and irritation [17]. Rosemary, a medicinal plant, is known for its medicinal properties, particularly in promoting hair growth and delaying gray hair onset. Its essential oil contains bioactive compounds like rosmarinic acid, ursolic acid, and carnosic acid, which improve scalp circulation and stimulate hair follicles. Rosemary oil also has antioxidant and anti-inflammatory properties, protecting hair from environmental damage and oxidative stress, thus promoting thicker, stronger hair and maintaining its natural color [18].

MelanoGray™ (Mibelle Biochemistry, Buchs, Switzerland) targets premature graying by enhancing melanin production. It is an ingredient that prevents premature graying by restoring hair's natural pigmentation. It stimulates melanin production, reactivating melanocytes, which are typically diminished due to aging or oxidative stress. This replenishes melanin cells and supports the hair growth cycle, improving scalp health and follicular integrity, making it an ideal hair revitalization and gray hair prevention solution [19].

AnaGain™ (Mibelle Biochemistry) is derived from pea sprouts and is known for stimulating hair growth by extending the anagen phase. It combats hair loss by targeting dermal papilla cells and reactivates hair growth by extending the growth phase and shortening the resting phase, increasing hair density and reducing shedding, supporting hair pigmentation, and delaying graying of hair [20].

This study evaluated the efficacy of Soulflower Rosemary Redensyl Hair Growth Serum, Tetragain™ (referred to as "test treatment" from here on), focusing on primary objectives such as changes in hair growth rate, hair length, anagen:telogen (A:T) ratio, hair density, thickness, hair fall count, and graying severity. The test treatment presents an innovative approach to hair care, addressing growth and pigmentation in one formulation by combining a range of the above scientifically backed ingredients into a single formulation, offering a comprehensive solution for both hair growth and pigmentation concerns. More details about the test product are given in the following section.

The study aimed to determine the test treatment serum's effectiveness in promoting hair growth, enhancing follicular activity, and reducing graying. Secondary objectives included assessing improvements in scalp health, specifically reductions in itchiness, redness, roughness, and scaliness. Additionally, a subjective consumer perception survey was conducted to evaluate user satisfaction, providing a comprehensive analysis of both clinical efficacy and participant-reported outcomes.

## Materials and methods

This was an open-label, single-arm, prospective, interventional, safety, efficacy, and tolerability study of the test treatment. The study was conducted at a contract research organization, NovoBliss Research Private Limited, Ahmedabad, India. The study protocol was approved by the ACEAS-Independent Ethics Committee on February 10, 2024 (approval number: NB240006-PT). The study was registered with the Clinical Trial Registry of India (CTRI) (registration number: CTRI/2024/02/062972). The clinical study treatment duration was 120 Days. Subject recruitment began on February 26, 2024, marked by the first subject's first visit, and concluded on August 8, 2024, with the last subject's last visit.

The study was carried out in compliance with the New Drugs and Clinical Trials Rules 2019 [21], ICH guidance E6 (R2) on 'Good Clinical Practice' [22], the Declaration of Helsinki (Brazil, October 2013), and the ICMR's National Ethical Guidelines for Biomedical and Health Research Involving Human Participants, 2017 [23]. Prior to being enrolled in the study, each participant signed an informed consent form. A thorough explanation of the study's goals, methods, confidentiality safeguards, and the voluntary nature of participation were all part of the consent process. All participating subjects' rights, safety, and well-being are protected by this thorough ethical framework, which guarantees that the study complies with national and international ethical norms.

Sample size and participants

The null hypothesis tested in this study was that there would be a reduction in hair density (cm²) after 90 days of applying the test treatment, while the alternative hypothesis proposed that hair density (cm²) would increase after the application of the test treatment. A paired comparison of hair density (per cm²) before and 90 days after applying the test treatment was conducted. To achieve approximately 80% power to detect a true mean change in hair density (cm²) from baseline to post-baseline, with a significance level (Type I error rate) of 5% (one-sided), the calculated effect size was 0.4689. The required sample size was determined to be 29.52, rounded up to 30 subjects to complete the study. Accounting for a 10% dropout rate, 34 subjects were enrolled to ensure that at least 30 subjects completed the study. A total of 32 healthy adult females aged 20 to 45 years with partially gray hair and complaints of hair fall were enrolled in the study, of which 30 completed the study and were analyzed. Tables 1, 2 give the exclusion and inclusion criteria.

**Table 1:** Inclusion criteria

Category	Criteria
General Health	Healthy, non-pregnant, non-lactating female individuals
Childbearing Potential	Women of childbearing potential included after self-reporting a negative pregnancy test.
Hair Condition	Partially gray hair, low hair growth, and self-reported complaints of hair thinning and loss.
Hormonal Contraception	Consistent use for at least six months prior and commitment to continue during the study.
Consent and Compliance	Provided written informed consent and committed to adhere to study protocols.
Restrictions	Avoidance of medicated/prescription shampoos, Minoxidil, other hair growth treatments, or hair dyes except the investigational treatment.

**Table 2:** Exclusion criteria

Category	Criteria
Hair Color	Excluded if any hair color other than gray.
Scalp Conditions	History of scalp dermatological conditions beyond hair loss or dandruff, scalp irritation, visible inflammation, or severe conditions.
Prior Hair Treatments	Hair growth procedures (transplants, laser treatments), topical treatments (within 4 weeks), or systemic treatments (within 3 months).
Current Hair Products	Use of marketed hair fall control or growth treatments during the study.
Hormonal/Drug Use	Chronic oral steroid use within 3 months prior to or during the study.
Other Clinical Trials	Participation in cosmetic, therapeutic, or hair/scalp care studies within the last 4 weeks.
Lifestyle Factors	Intention to shave scalp during the study.

Objectives

The primary objectives were to evaluate the effectiveness of the test treatment in terms of changes in hair growth rate, A:T ratio, hair density, hair thickness, hair fall counts, and Graying Severity Score (GSS) [24]. The secondary objectives were to evaluate the effectiveness of the test treatment in terms of changes in scalp appearance, i.e. itchiness, redness, roughness, and scaliness of scalp. Additionally, consumer perception of the test treatment was evaluated using a subjective questionnaire (see Appendices).

Study procedure

The study procedure is given in Table 3. The CASLite Nova Hair Analysis System (Catseye Systems & Solutions Pvt Ltd, Navi Mumbai, Maharashtra, India) is an advanced, patented, software-based technique designed to accurately measure and analyze hair growth rate, hair thickness, hair density, and scalp condition. Standardized and validated methods for assessing hair growth rate, hair length, anagen-to-telogen ratio, hair density, and hair thickness have been utilized to evaluate the efficacy of the test treatment [25]. In the study, evaluations were performed four days prior to Day 01, and subsequently on Day 1, Day 45, Day 87, Day 90, and Day 120, providing a comprehensive analysis over time.

**Table 3:** Visits and description

Visit	Day	Description
Visit 01	4 days prior	Screening of participants.
Visit 02	Day 01	Enrollment, baseline evaluations, and commencement of test treatment usage.
Visit 03	Day 45	Mid-treatment evaluation while participants continued using the test treatment.
Visit 04	Day 87	Ongoing evaluation during treatment usage.
Visit 05	Day 90	Additional evaluation while participants continued treatment.
Visit 06	Day 120	Final evaluations marking the conclusion of the study.

Dermatological evaluations to assess the safety and efficacy of the test treatment on the scalp and hair were carried out by a qualified dermatologist, using both visual and tactile examination methods. Key parameters evaluated included scalp health, signs of irritation or adverse reactions (such as redness, itching, or flaking), and overall hair condition, including texture, shininess, and strength. The severity of graying was assessed by the dermatologist using a standardized scoring scale, GSS [24]. The evaluation classified the severity into three distinct categories: mild, moderate, and severe. This categorization allowed for a systematic assessment of graying severity, providing a reliable and clinically relevant measure for the study's analysis. Special attention was given to the presence of any allergic reactions or unwanted side effects. The dermatologist also monitored hair density and hair loss patterns to track any changes during the course of the study, ensuring a comprehensive evaluation of the treatment’s impact on both the scalp and hair. Skin conditions such as redness, roughness, and itchiness were measured by the Scalp Photographic Index (SPI) [26].

Test product 

The Soulflower Rosemary Redensyl Hair Growth Serum (Tetragain™) is designed for hair growth and anti-gray hair treatment. Marketed by Soulflower, a brand under PT Invent India Pvt Ltd, it is manufactured by Bo International (Gurugram, Haryana, India). The serum is directly sprayed onto a clean scalp and massaged thoroughly. There’s no need to rinse and hair can be styled as usual. For optimal results, the test product was applied once a day for a period of 120 days on the scalp of the volunteers. The formula contains a blend of active ingredients, including AnaGain, Redensyl, *Oryza sativa* water extract, *Salvia hispanica* seed extract, MelanoGray, *Rosmarinus Officinalis* oil, caffeine, keratin, biotin, and *Melaleuca alternifolia* oil, all intended for topical application.

Data and statistical analysis

All data were reviewed before analysis to ensure accuracy and completeness. Frequency analyses and cross-tabulations were performed to ensure data accuracy and consistency. Missing data were addressed through appropriate imputation methods or excluded from the analysis, depending on the extent and nature of the missingness. The results of the statistical tests evaluated by the paired t-test, including p-values, were reported with corresponding confidence intervals to provide a measure of precision and reliability.

Continuous variables were summarized using descriptive statistics, including sample size (N), mean, standard deviation (SD), median, minimum, and maximum values. Categorical variables were presented as frequencies and percentages, with graphical representations provided where applicable. Statistical analysis was conducted using IBM SPSS Statistics for Windows, Version 29.0.1.0 (Released 2023; IBM Corp., Armonk, New York, United States) and Microsoft Excel 2019 (Microsoft Corporation, Redmond, Washington, United States), applying a 5% level of significance. Subjects who were withdrawn from the study were excluded from the statistical analysis.

## Results

Primary endpoints

Demographic and Other Baseline Characteristics

**Figure 1:**
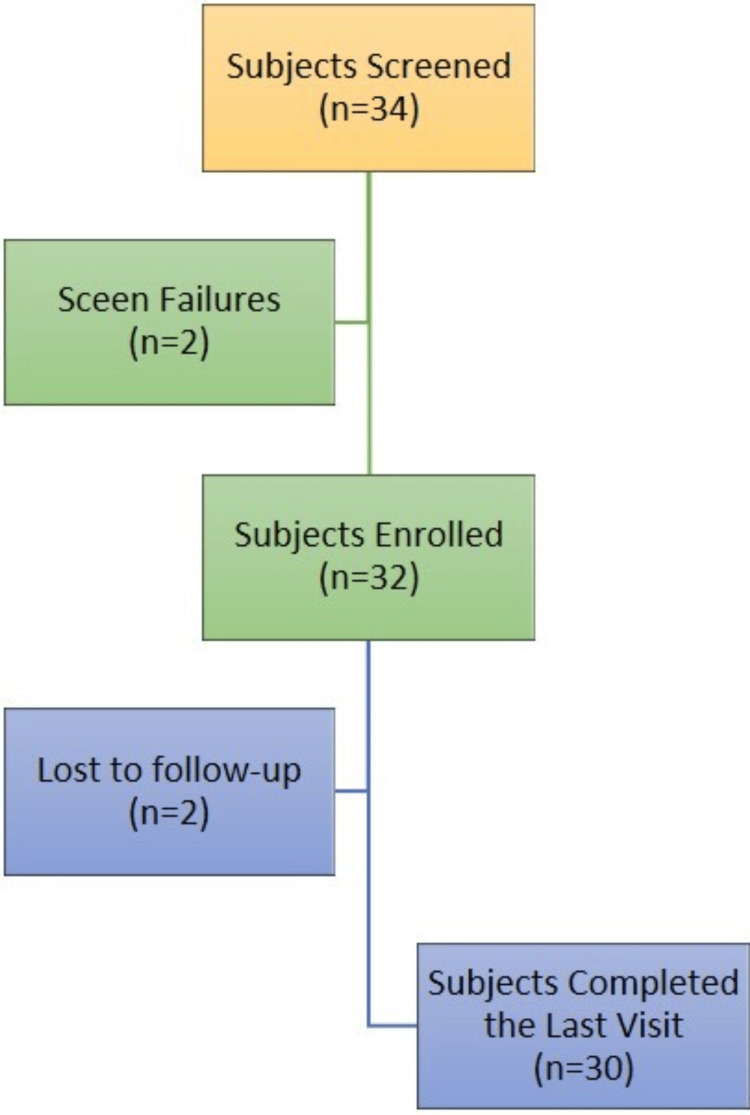
Flowchart denoting the screening and inclusion of participants

A total of 32 female subjects, aged 20-45 years, were enrolled in the study, with 30 completing it (Figure 1). Despite two dropouts, complete data was collected for both pre- and post-hair growth and anti-gray hair assessments, ensuring robust statistical analysis. The study maintained high compliance with the intervention and assessment schedules. The mean age of the participants was 37.87 ± 4.94 years. Additionally, the mean height was 157.47 ± 6.45 cm, and the mean weight was 62.45 ± 12.55 kg. 

Hair Growth Rate Assessment Through Phototrichogram using CASLite Nova

**Figure 2:**
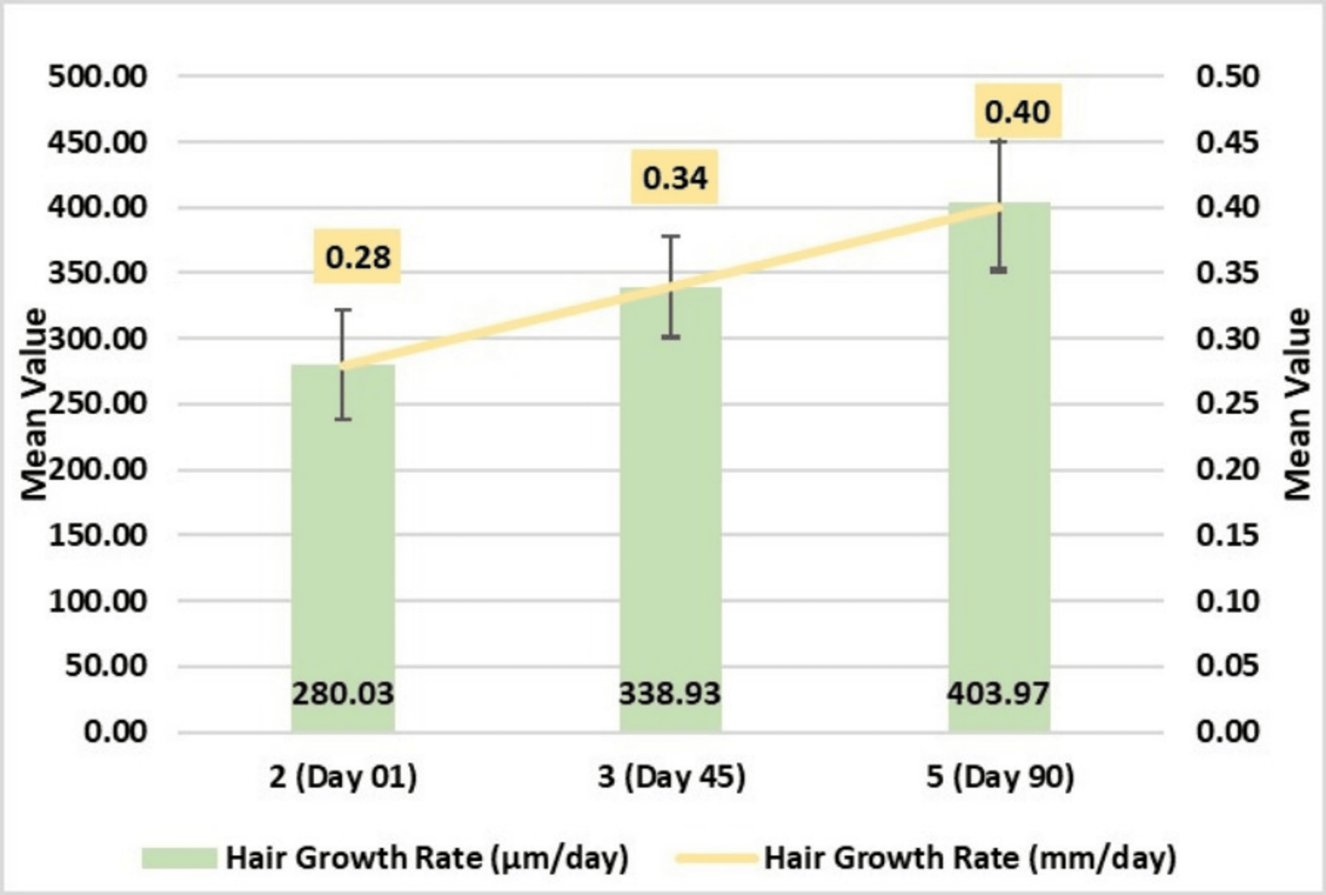
Change in hair growth rate (μm/day and mm/day) assessed by CASLite Nova* *Catseye Systems & Solutions Pvt Ltd, Navi Mumbai, Maharashtra, India

Hair growth rate assessment through phototrichogram using CASLite Nova demonstrated a significant enhancement throughout the study. Results indicated a significant increase in hair growth rate from baseline, with a notable 338.93 ± 38.47 μm/day, which was statistically significant (p-value <0.0001) on Day 45. This effect was further intensified by Day 90 when a 403.97 ± 49.72 μm/day improvement was recorded. These findings underscore the substantial impact of the test treatment in promoting hair growth rate, with a highly significant p-value of <0.0001 (Figure 2).

Hair Length Assessment Through Phototrichogram using CASLite Nova

**Figure 3:**
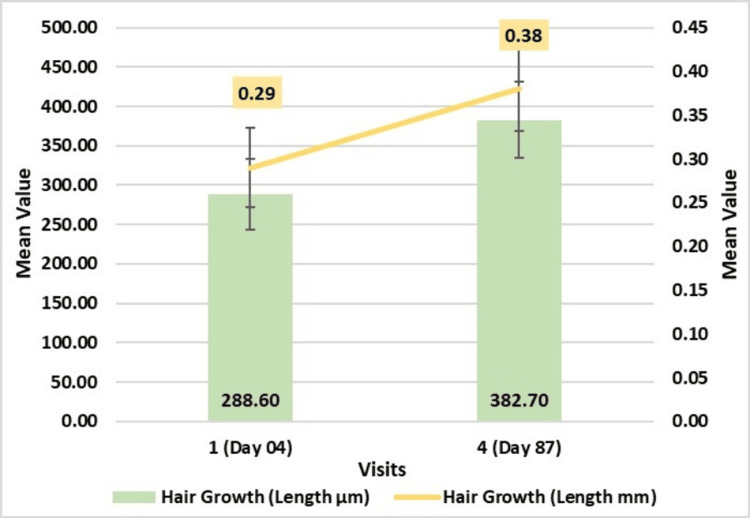
Change in hair length (μm and mm): tattoo method assessed by CASLite Nova* *Catseye Systems & Solutions Pvt Ltd, Navi Mumbai, Maharashtra, India

Hair length assessment through phototrichogram using CASLite Nova demonstrated a significant enhancement throughout the duration of the study. Hair length increased significantly by 382.70 ± 48.28 μm by Day 87 (p < 0.001) (Figure 3).

A:T Ratio Assessment Through Phototrichogram Using CASLite Nova

**Figure 4:**
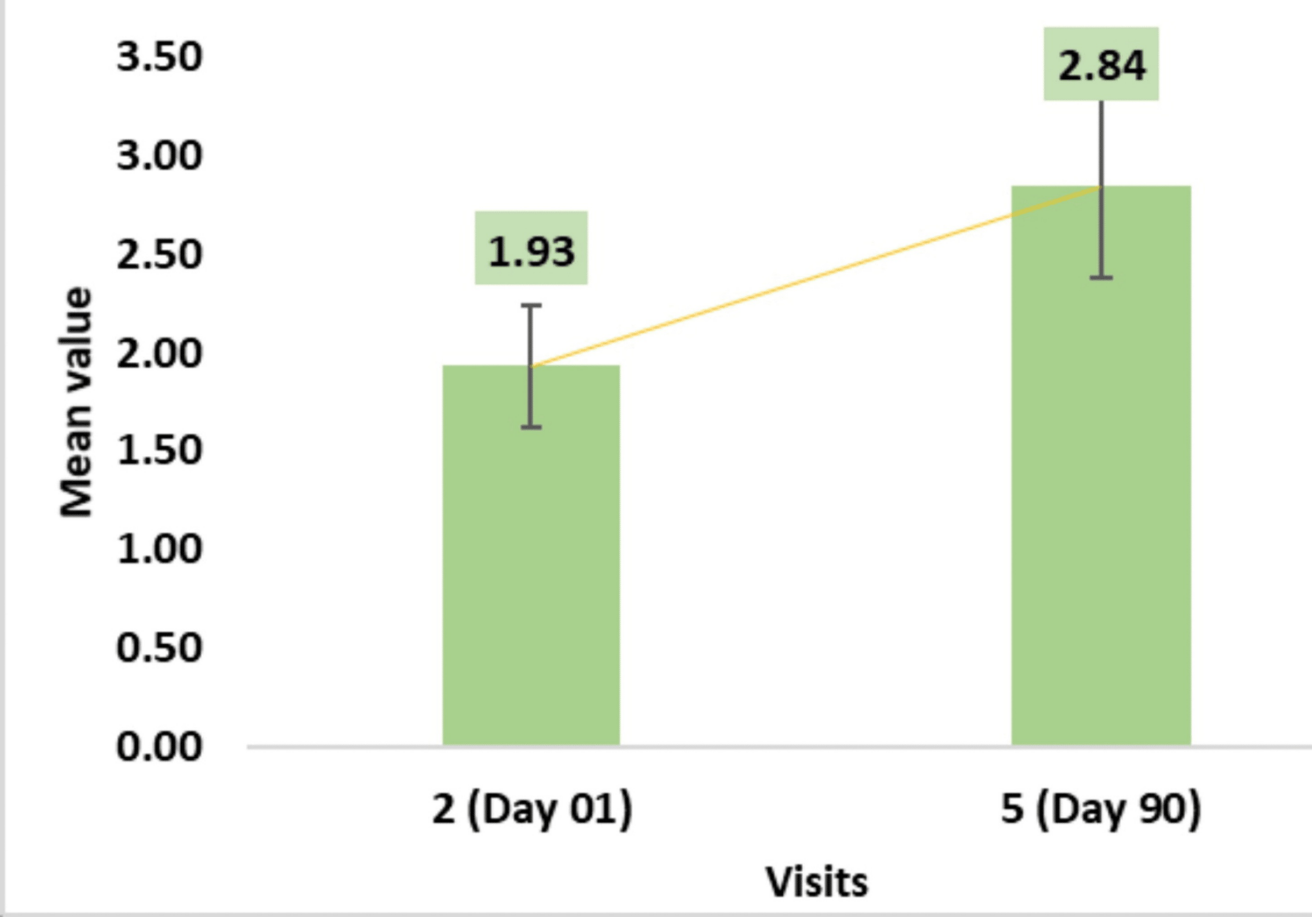
Change in A:T ratio: phototrichogram assessed by CASLite Nova* *Catseye Systems & Solutions Pvt Ltd, Navi Mumbai, Maharashtra, India

A:T ratio assessment through phototrichogram using CASLite Nova demonstrated significant improvement from baseline to post-usage of the test treatment, with a 2.84 ± 0.46 increase by Day 90 (Figure 4).

Assessment of Hair Density Through Phototrichogram Using CASLite Nova

Hair density assessment through phototrichogram using CASLite Nova demonstrated statistically significant enhancement in hair density (cm^2^) following the application of test treatment when compared with the baseline evaluation. Hair density improved to 265.89 ± 33.39 cm^2^ on Day 45, 315 ± 31.66 cm^2^ on Day 90, 335.33 ± 28.52 cm^2^ on Day 120 with highly significant p-value of <0.001.

Assessment of Hair Thickness Through Phototrichogram Using CASLite Nova

**Figure 5:**
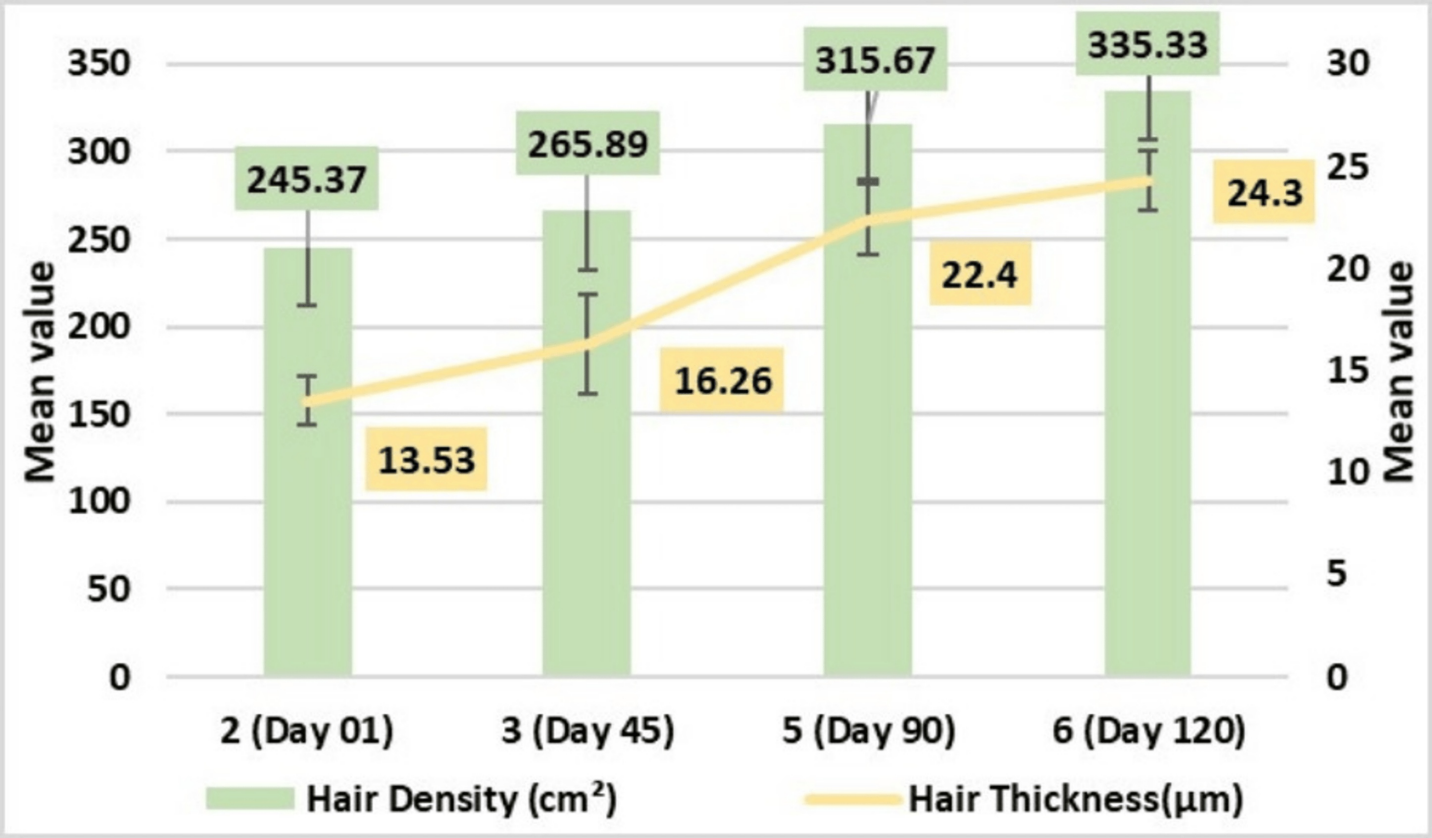
Change in hair density (cm²) and hair thickness (µm) assessed by CASLite Nova* *Catseye Systems & Solutions Pvt Ltd, Navi Mumbai, Maharashtra, India

Hair thickness assessment through phototrichogram using CASLite Nova demonstrated statistically significant enhancement in hair thickness (μm) following the application of test treatment when compared with the baseline evaluation. Hair thickness showed improvement of 16.26 ± 2.43 μm on Day 45, 22.40 ± 1.71 μm on Day 90, and 24.30 ± 1.51 μm on Day 120. These results were statistically highly significant with a p-value of <0.001 (Figure 5).

Assessment of Hair Fall Without Bulb Count Using the 60-Second Hair Comb Test 

The number of hair that fell without bulbs using a 60-second hair comb test demonstrated a significant reduction in count following the application of test treatment compared to baseline measurements. The reduction was consistent across the evaluation period with 15.89 ± 8.80 reduction on Day 30, 12.20 ± 7.69 reduction on Day 90, and 7.60 ± 4.34 reduction on Day 120; the findings were statistically highly significant (p-value <0.001).

Assessment of Hair Fall Count With Bulb Using the 60-Second Hair Comb Test

The number of hair that fell with the bulb using the 60-second hair comb test demonstrated a significant reduction in count following the application of test treatment compared to baseline measurements with 17.22 ± 9.63 reduction on Day 30, 11.23 ± 5.41 reduction on Day 90, and 6.97 ± 3.01 reduction on Day 120. These results are statistically highly significant with a p-value of <0.001.

Assessment of Total Hair Fall Using the 60-Second Hair Comb Test

**Figure 6:**
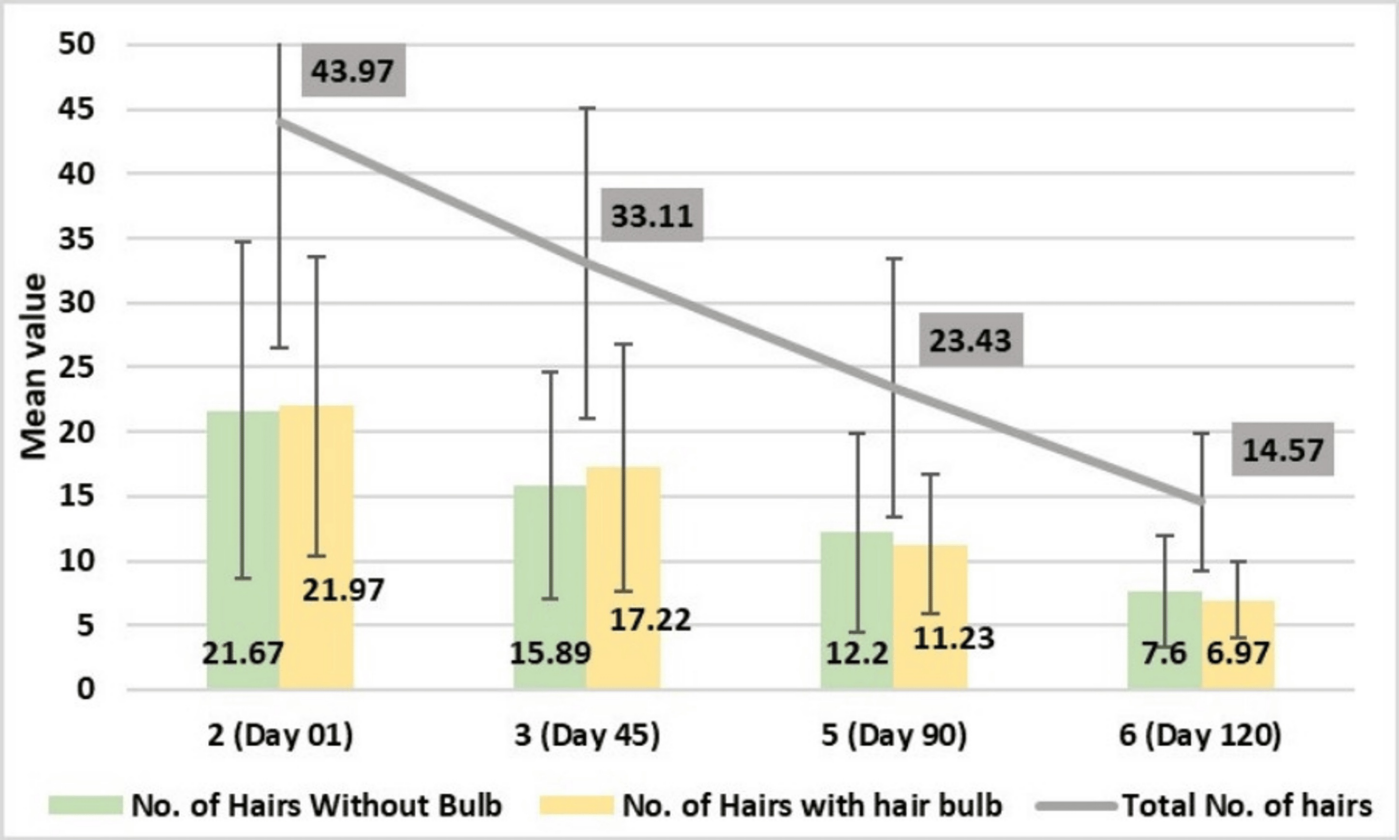
Change in numbers of hair fallen with bulb and without bulb, and total number of hair fall assessed by the 60-second hair comb method

The total number of hair that fell using the 60-second hair comb test demonstrated a significant reduction in count following the application of test treatment compared to baseline measurements, with 33.11 ± 12.02 reduction on Day 30, 23.43 ± 9.97 reduction on Day 90, and 14.57 ± 5.32 reduction on Day 120. These results were statistically highly significant with p-values of <0.001 (Figure 6).

GSS Assessed by Phototrichogram Using CASLite Nova

**Figure 7:**
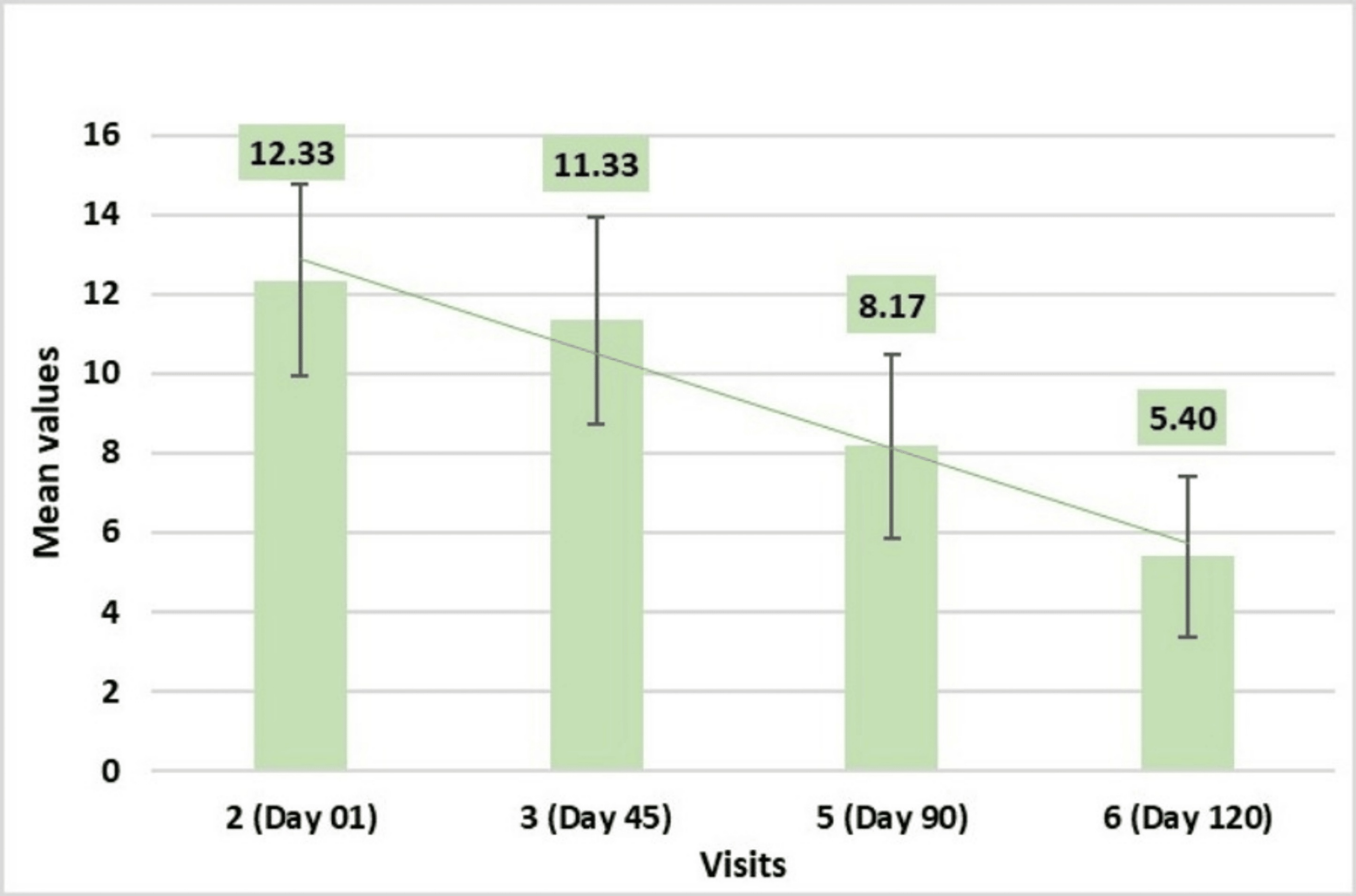
Change in Gray Severity Score (GSS) assessed by CASLite Nova* *Catseye Systems & Solutions Pvt Ltd, Navi Mumbai, Maharashtra, India

GSS assessed by phototrichogram using CASLite Nova demonstrated a statistically significant reduction following the administration of the test treatment, compared to baseline measurements. Throughout the study, reductions were observed: 11.33 ± 2.60 reduction on Day 30 indicating severe gray hairs, 8.27 ± 2.23 reduction on Day 90 indicating moderate gray hairs, and 6.53 ± 2.01 reduction on Day 120 indicating moderate gray hairs. These results highlight the significant impact of the test treatment on reducing the GSS, with the findings being highly statistically significant (p-values <0.001) (Figure 7).

Secondary endpoints

General Appearance of Scalp 

The scalp conditions were assessed by the SPI.

Scalp itchiness: A total of 18 (60%) subjects had mild itchiness and 12 (40%) had moderate itchiness on Day 1 before usage of the test treatment. On Day 45, 10 (37.04%) subjects had no itchiness, 16 (59.26%) had mild itchiness, and one (3.70%) had moderate itchiness. On Day 90, 23 (76.67%) subjects had no itchiness and seven (23.33%) had mild itchiness. On Day 120, 30 (100%) subjects had no itchiness.

Skin redness: A total of 18 (60%) subjects had no redness and 12 (40%) subjects had mild redness on Day 01 before usage of the test treatment. On Day 45, 30 (100%) subjects had no redness.

Skin roughness: A total of two (6.67%) subjects had no roughness, 15 (50%) had mild roughness, and 13 (43.33%) had moderate roughness on Day 1. On Day 45, 10 (37.04%) subjects had no roughness, 16 (59.26%) had mild roughness, and one (3.70%) had moderate roughness. On Day 90, 17 (56.67%) subjects had no roughness and 13 (43.33%) had mild roughness. On Day 120, 30 (100%) subjects had no roughness.

Skin scaliness: A total of 26 (86.67%) subjects had mild scaliness and four (13.33%) had moderate scaliness before usage of the test treatment on Day 1. On Day 45, six (22.22%) subjects had no scaliness, 20 (74.07%) subjects had mild scaliness, and one (3.70%) subject had moderate scaliness. On Day 90, 14 (46.67%) subjects had no scaliness, 16 (53.33%) had mild scaliness, and on Day 120, 30 (100%) of the subjects had no scaliness.

Subjects' Perceptions Questionnaire

The Subjects' Perceptions Questionnaire included questions assessing the subjects' perceptions of the effectiveness of the test treatment in areas such as redness, dryness, itchiness, and burning sensation of the scalp upon the usage of the test treatment, whether hair becomes soft, silky, and shiny, effectiveness in the reduction of gray hair, improvement of hair thickness and density, reduction in hair fall, and satisfaction after usage of test treatment.

**Figure 8:**
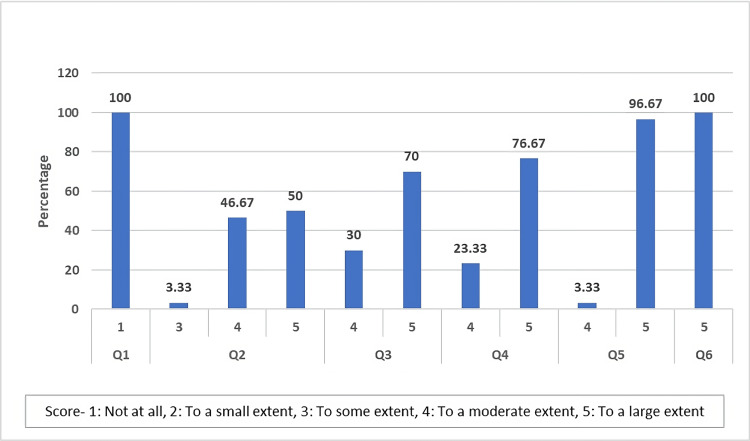
Distribution of responses to the Consumer Perception Questionnaire according to scores Q1: Did you experience any irritation reactions such as redness, dryness, itchiness, burning sensation, etc of the scalp upon usage of test product? Q2: Do you feel that your hair becomes soft, silky and shiny after usage of test product? Q3: Do you feel the test product is effective in the reduction of gray hair? Q4: Do you feel the test product is effective in improving hair thickness and density? Q5: Do you feel the test product is effective in reducing hair fall? Q6: How much are you satisfied with the usage of test product?

The study found that two (6.66%) subjects had used another treatment before, while 28 (93.33%) had not. On Day 1, two (6.66%) subjects experienced irritation reactions like redness, dryness itchiness, and burning sensation, while 28 (93.33%) did not. On Day 120, all 30 (100%) subjects reported not experiencing any irritation reactions. On Day 120, 30 (100%) of subjects experienced soft, silky, and shiny hair, with one (3.33%) experiencing this to some extent, four (46.67%) to a moderate extent, and 15 (50.00%) to a large extent. On all Day 120, 30 (100%) subjects reported a reduction in gray hair with nine (30%) to a moderate extent, and 21 (70%) to a large extent. On Day 120, all 30 (100%) subjects reported an improvement in hair thickness and density, with seven (23.33%) to a moderate extent and 23 (76.67%) to a large extent. On Day 120, one (3.33%) subject experienced a reduction in hair fall to a moderate extent and 29 (96.67%) to a large extent. On Day 120, all 30 (100%) subjects reported being satisfied with the treatment to a large extent (Figure 8).

Photographic assessment

**Figure 9:**
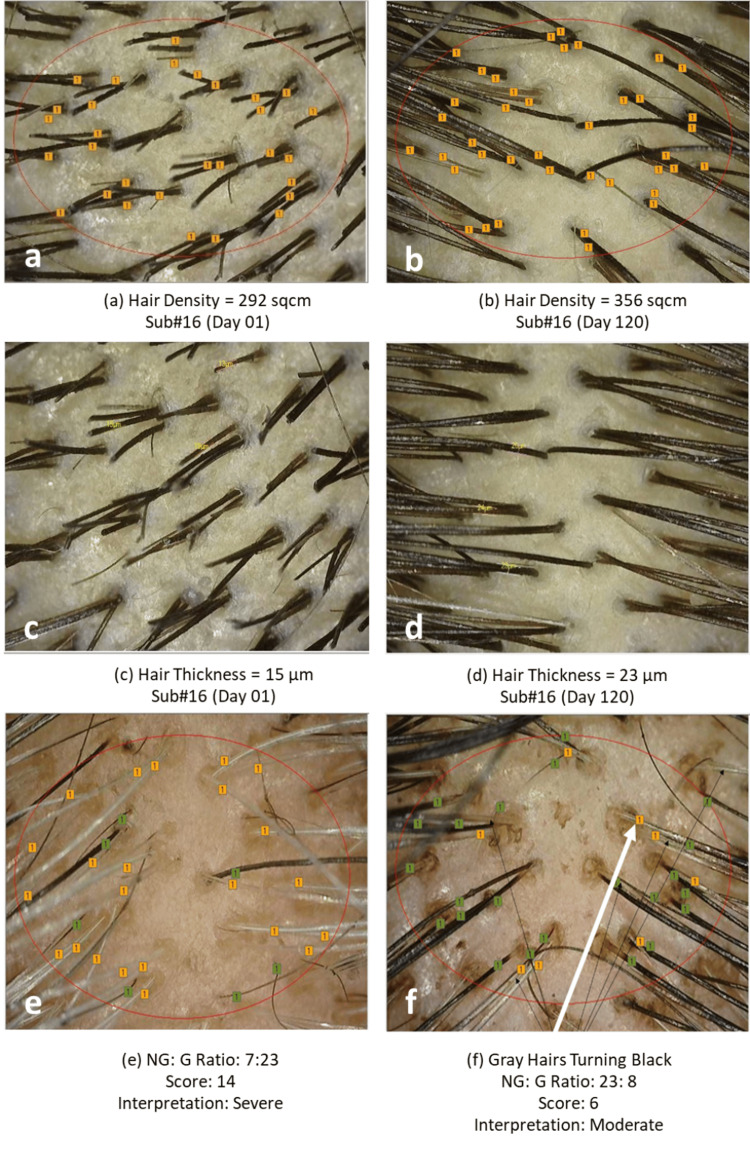
Change in (a,b) hair density, (c,d) hair thickness, and (e,f) GSS as assessed by CASLite Nova in Subject 16 GSS: Graying Severity Score; NG: non-gray; G: gray

**Figure 10:**
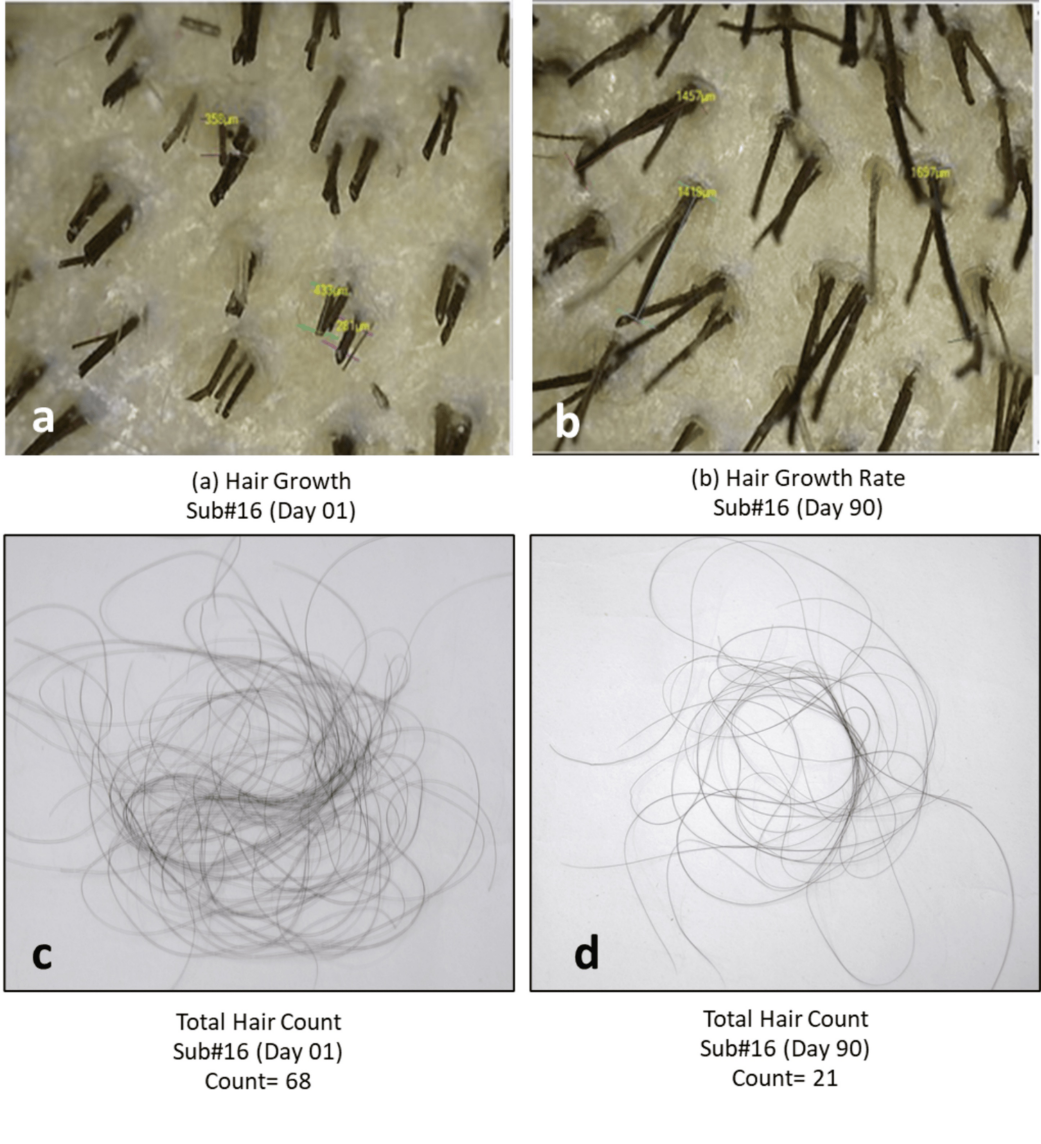
Change in (a,b) hair growth rate and (c,d) total hair fall count as assessed by CASLite Nova and the 60-second hair comb test in Subject 16

In the study, hair growth and gray hairs were evaluated using a detailed photographic assessment. High-resolution images of affected scalp areas were captured under standardized conditions to ensure consistency across all participants. These images were then documented to assess the extent and severity of hair loss and gray hair over time (Figures 9, 10)

## Discussion

Alopecia is a multifactorial disorder characterized by partial or complete hair loss, affecting both the scalp and other areas of the body. The pathophysiology of alopecia varies depending on its type; AGA is primarily driven by genetic predisposition and androgen dysregulation, while alopecia areata (AA) involves autoimmune mechanisms, where autoreactive T cells target hair follicles. Dysregulation in the hair growth cycle, particularly an altered balance between the anagen (growth) and telogen (resting) phases, contributes to hair thinning and loss [27].

Current therapeutic strategies for alopecia aim to modulate these underlying mechanisms. Topical minoxidil and oral finasteride remain the mainstay treatments for AGA, with both showing efficacy in promoting hair regrowth by prolonging the anagen phase and reducing follicular miniaturization. In AA, immunosuppressive agents such as corticosteroids and emerging Janus kinase (JAK) inhibitors have shown promise in reversing hair follicle autoimmunity. However, despite advancements in treatment options, a comprehensive understanding of alopecia’s molecular basis remains crucial for developing targeted and effective therapies [28].

Several ingredients have shown positive effects on hair growth and health. AnaGain, for example, revitalizes hair follicles to stimulate growth and increase density [20]. Redensyl strengthens hair and combats hair loss by targeting hair stem cells and the dermal papilla [13]. *Oryza sativa* water extract promotes scalp health and enhances hair strength [14], while *Salvia hispanica* seed extract helps reduce breakage [15], fostering stronger hair. MelanoGray supports melanin production and reduces oxidative stress in hair follicles, effectively decreasing the presence of gray hair [19]. Additionally, rosemary oil is known for promoting hair growth and preventing hair loss, contributing to overall scalp health [18]. Collectively, these ingredients offer a comprehensive approach to enhancing hair health, density, and pigmentation.

In this study, the test treatment (Soulflower Rosemary Redensyl Hair Growth Serum, Tetragain™) was evaluated for efficacy in addressing key parameters related to hair health, including hair growth, graying, density, and thickness. According to the literature survey, the normal hair growth rate for a healthy scalp is 0.4 mm/day [29]. In our study, subjects with hair fall complaints and having hair growth rate mean of 0.2 mm/day were enrolled and results showed significant improvement in growth at Day 45 with 0.3 mm/day and 0.40 mm/day at Day 90. There was a significant improvement in hair thickness, which is particularly noteworthy, as thicker hair is often perceived as healthier and more robust. Furthermore, there was a reduction in hair fall indicating a potential stabilization of the hair growth cycle, which is essential for maintaining a fuller appearance. The observed enhancement in the GSS underscores the treatment's potential not only to promote hair growth but also to address concerns related to premature graying. Importantly, the absence of adverse effects throughout the study period suggests that the test treatment is safe for use. The high satisfaction rate among subjects reflects the perceived effectiveness of the treatment, highlighting its potential as a viable option for individuals seeking to improve hair health and manage gray hair effectively. Overall, these findings support the premise that the test treatment could offer significant benefits in promoting hair vitality and addressing common hair-related concerns.

Rosemary’s role in enhancing scalp circulation and inhibiting 5-alpha-reductase supports healthy hair growth, making it a valuable natural alternative to chemical treatments for improving hair health and appearance [30]. Several studies have highlighted rosemary’s effectiveness in promoting hair health through its antioxidant, anti-inflammatory, and circulation-boosting properties. In the present study, the rosemary-based hair serum demonstrated its ability to nourish the scalp and stimulate hair follicles, with no adverse reactions such as redness or itching.

Bansal and Bansal demonstrated that a photo ingredient-based solution containing procapil, capixyl, Redensyl, bicapnil, and AnaGain, applied at a dose of 1 ml twice daily, demonstrated efficacy and safety in the long-term management of MPHL and FPHL [31]. The treatment significantly enhanced scalp hair appearance, improving hair density, strength, and thickness without any reported adverse effects. These findings suggest that this formulation can be used as either a monotherapy or an adjunct to conventional treatments for hair loss. 

Limitations and recommendations

A significant limitation of this hair study is its single-arm, open-label design, where all participants received the test treatment without a comparison group, such as a placebo. The absence of a control group makes it challenging to determine whether observed effects, such as hair growth or reduction in gray hair, are due to the treatment itself or other factors like natural hair cycles or placebo effects. The open-label format also introduces potential bias, as both participants and researchers were aware of the treatment being administered. Without blinding or randomization, the strength of the findings is reduced. Future randomized controlled trials (RCTs) would provide more robust evidence of the treatment’s efficacy. Additionally, the study’s small sample size and relatively short duration of 120 days may limit the generalizability and long-term applicability of the results. Other important factors, such as environmental influences, dietary habits, genetic predispositions, and hormonal imbalances, were not accounted for, which could impact the outcomes. Long-term studies are necessary to better understand the sustained benefits of the treatment with continued use.

Despite these limitations, the treatment shows potential as a treatment for both hair growth and gray hair reduction. The combination of ingredients like MelanoGray, rosemary oil, Redensyl,* Oryza sativa* water extract, and *Salvia hispanica *seed extract appears to stimulate hair growth while addressing gray hair. Future research should build on these findings by assessing the long-term efficacy and safety of the treatment in more diverse populations. Further exploration of the treatment’s effects in combination with lifestyle and environmental factors could also provide additional insights into optimizing hair treatment strategies.

## Conclusions

The clinical safety and efficacy study of Soulflower Rosemary Redensyl Hair Growth Serum (Tetragain™) demonstrated its safety and effectiveness for women aged 20-45 years who were experiencing hair loss and premature graying. The serum combines potent active ingredients, including AnaGain, Redensyl, rice water extract, chia seed extract, MelanoGray, and rosemary oil, which work synergistically to promote hair growth, strengthen hair, enhance scalp health, reduce hair loss, and address premature graying by stimulating melanin production and reducing oxidative stress. The formulation also leverages the antioxidant and antimicrobial benefits of rosemary oil for overall scalp care.

The synergistic effect of these bioactive components normalizes the disrupted hair growth cycle, fostering a more balanced and healthier process, specifically addressing the underlying causes of hair thinning and loss and maintaining natural hair color, making this serum an effective choice for individuals looking to delay premature graying. There were no adverse reactions reported, confirming its appropriateness for regular use in a daily hair care regimen aimed at enhancing hair density, reducing hair fall, and addressing early signs of graying in women. 

## Appendices

**Table 4:** Consumer perception questionnaire

Sr. No	Questionnaire	Response
1	Did you experience any irritation reactions such as redness, dryness, itchiness, burning sensation, etc of the scalp upon usage of test product?	1 = Not at all; 2 = To a small extent; 3 = To some extent; 4 = To a moderate extent; 5 = To large extent
2	Do you feel that your hair becomes soft, silky and shiny after usage of test product?	1 = Not at all; 2 = To a small extent; 3 = To some extent; 4 = To a moderate extent; 5 = To large extent
3	Do you feel the test product is effective in the reduction of gray hair?	1 = Not at all; 2 = To a small extent; 3 = To some extent; 4 = To a moderate extent; 5 = To large extent
4	Do you feel the test product is effective in improving hair thickness and density?	1 = Not at all; 2 = To a small extent; 3 = To some extent; 4 = To a moderate extent; 5 = To large extent
5	Do you feel the test product is effective in reducing hair fall?	1 = Not at all; 2 = To a small extent; 3 = To some extent; 4 = To a moderate extent; 5 = To large extent
6	How much are you satisfied with the usage of test product?	1 = Not at all; 2 = To a small extent; 3 = To some extent; 4 = To a moderate extent; 5 = To large extent
